# Plant-mediated nickel oxide nanoparticles show species-dependent antibacterial, antioxidant, anti-inflammatory and antidiabetic activities

**DOI:** 10.1038/s41598-025-15951-4

**Published:** 2025-08-24

**Authors:** Alaa Hassan Said, Fatma. Shaibah, M. Moustafa, Rokaia B. Elamary

**Affiliations:** 1 Electronics and Nano Devices Lab, Faculty of Science, South Valley University, Qena, Egypt; 2 Physics Department, Faculty of Science Qena, South Valley University, Qena, Egypt; 3 Physics Department, Faculty of Science, Minia University, Minia, Egypt; 4 Botany and Microbiology Department, Faculty of Science, Luxor University, Luxor, Egypt

**Keywords:** Green synthesis, Nickel oxide nanoparticles, Anticancer, Antibacterial, Antibiofilm, Anti-inflammatory, Antioxidant and Antidiabetic, Biophysics, Biotechnology, Nanoscience and technology

## Abstract

Nickel oxide nanoparticles are renowned for their diverse properties, including magnetic, electrical, optical, and catalytic capabilities, making them highly suitable for both industrial and biomedical applications. This study synthesized nickel oxide nanoparticles using both chemical and green synthesis methods with extracts from four plant species: *Medicago sativa* L., *Euphorbia milii* Des Moul., *Codiaeum variegatum* (L.) A. Juss*.*, and *Helianthus annuus* L. All samples had consistent face-centered cubic structures, as proven by X-ray diffraction. However, electron microscopy and Fourier-transform infrared spectra showed that the green-synthesized particles had tighter size distributions and unique surface functions due to the presence of plant phytochemicals. According to in vitro tests, *Codiaeum variegatum* mediated nanoparticles showed the strongest multifunctional bioactivity, including increased scavenging of free radicals, targeted cytotoxicity against A549 lung cancer cells, and substantial inhibition of cyclooxygenase-1 and important enzymes that hydrolyze carbohydrates. Antibacterial tests demonstrated that *Staphylococcus aureus* was efficiently reduced by both chemically produced and *Codiaeum variegatum* derived nanoparticles, whereas biofilm experiments shown that *Medicago sativa* derived nanoparticles caused greater disruption. These findings demonstrate that the performance of nanoparticles is controlled by their botanical source, allowing NiO nanoparticles to be tailored for specific medicinal uses. Our results open the door for additional in vivo testing and process optimization while validating green synthesis as an environmentally responsible method of producing NiO nanoparticles with adjustable bioactivities.

## Introduction

Nickel oxide NPs (NiO NPs) have garnered considerable interest recently in many applications due to their distinctive physicochemical properties. As a p-type semiconductor with a wide band gap ranging from 3.6 to 4.0 eV, NiO NPs proves excellent thermal stability, optical transparency, magnetic responsiveness, and catalytic efficiency. These properties make them ideal for a wide range of industrial uses, such as solar energy conversion, battery technology, electrochromic systems, gas detection, and supercapacitor development^1^. Because of their surface tunability, biocompatibility, and capacity to generate reactive oxygen species (ROS) that can interact with microbial or malignant cells, NiO NPs have demonstrated increasing promise in biomedical applications^2^.

Conventional synthesis methods, like sol–gel, hydrothermal, and chemical precipitation, frequently need significant energy inputs, hazardous solvents, and severe reaction conditions. Green synthesis, on the other hand, employing plant extracts, offers a sustainable, economical, and environmentally beneficial substitute. As natural reducing and capping agents, the phytochemicals (such as flavonoids, terpenoids, saponins, and alkaloids) in these extracts guide the nucleation and development of NPs while improving biological functionality^3,4^. The selection of plant species affects the size, shape, surface charge, and eventually bioactivity of the particles, enabling customized creation for certain medicinal uses^5,6^.

Several studies have demonstrated the powerful anticancer properties of plant-derived NiO NPs. NiO NPs derived from *Moringa oleifera* leaves showed considerable cytotoxicity against human colorectal cancer (HT-29) cells, with dose-dependent suppression of cell growth^7^. Similarly, NiO NPs synthesized from *Aegle marmelos* displayed effective anticancer activity against A549 lung carcinoma cells^8^, whereas *Callistemon viminalis* floral extract-derived NiO NPs were reported to be efficacious against HepG2 liver cancer cells^9^. The plant extracts’ phytochemical contents are largely responsible for the increased generation of ROS, mitochondrial dysfunction, and caspase-dependent apoptosis that result in cellular death. Green NiO NPs also showed excellent antibacterial properties. NiO NPs synthesized from *Abutilon indicum* leaf extract shown broad-spectrum antibacterial action, effectively suppressing both Gram-positive and Gram-negative bacterial strains^10^. *Callistemon viminalis* mediated NiO NPs showed inhibitory effects against *Klebsiella pneumoniae* and *Proteus vulgaris*, with MICs as low as 12.5 µg/mL^9^. Additionally, NiO NPs derived from *olive leaf* extract were found to be very efficient against both *Candida albicans* and *Hyalomma dromedarii ticks*, expanding their potential for antifungal and antiparasitic therapy^11^.

Aside from their antibacterial and anticancer properties, biogenic NiO NPs have displayed notable anti-inflammatory and antioxidant properties. NiO NPs synthesized from *Abutilon indicum* exhibited potent free radical scavenging properties, indicating their potential application in the treatment of oxidative stress-related disorders^10^. Similarly, *Rhamnus triquetra* derived NiO NPs demonstrated strong antioxidant properties, which are thought to be amplified by the extract polyphenolic components^12^. These findings highlight the double role of plant extracts, which not only facilitate the formation of NiO NPs but also improve their therapeutic action. A key mechanistic role is played by the plant extracts used in this synthesis process. These extracts, which are rich with flavonoids, phenols, terpenoids, and alkaloids, serve as capping agents, stabilizing the surface of NPs and avoiding agglomeration while also reducing Ni^2+^ ions into NiO. Cellular absorption, bioavailability, and interaction with biological targets are all critically dependent on the surface chemistry of the produced NPs, which is directly influenced by these biomolecules^3,13^. Furthermore, each plant has distinctive phytochemical signatures that provide unique biological profiles for the resulting NPs. The potential use of green-synthesized NiO NPs in a variety of therapeutic domains, including oncology, infectious disease management, and disorders linked to oxidative stress, is highlighted by their multifunctional characteristics, which include anticancer, antimicrobial, antioxidant, and anti-inflammatory activities. This emphasizes the vital significance of choosing suitable plant sources according to the NPS intended industrial or biomedical applications.

Despite these promising attributes, some difficulties still exist. Additional optimization is needed to scale up green synthesis while preserving batch-to-batch uniformity. Comprehensive in vivo safety profiles and long-term NPs stability have not yet been thoroughly explored, and further research is necessary to determine the exact molecular mechanisms by which various plant extracts alter bioactivity. Overcoming these limitations will be critical for translating green-synthesized NiO NPs into clinical and industrial applications. In light of these considerations, this study aims to explore the influence of four plant species (*Medicago sativa* L., *Euphorbia milii* Des Moul., *Codiaeum variegatum* (L.) A. Juss., and *Helianthus annuus* L.) on the green synthesis of NiO NPs. We thoroughly assess their physicochemical characteristics and a wide range of biological activities, such as their ability to inhibit α-amylase and α-glucosidase for antidiabetic effects, their antioxidant capacity, their antibacterial efficacy, their antibiofilm activity, their COX-1-mediated anti-inflammatory potential, and their cytotoxicity against A549 lung carcinoma cells.

## Materials and methods

### Chemicals and reagents

All chemicals and reagents used in this study were of analytical grade and utilized without further purification. Nickel(II) acetate tetrahydrate (Ni(CH_3_COO)_2_·4H_2_O, ≥ 98%) and sodium hydroxide (NaOH, ≥ 99%) were obtained from Alfa Aesar, USA. Ethanol (≥ 99.8%), propanol (≥ 99.5%), acarbose (≥ 98%), 3,5-dinitrosalicylic acid (DNSA, ≥ 98%), bovine serum albumin (≥ 98%), diclofenac sodium (≥ 98%), and dimethyl sulfoxide (DMSO, ≥ 99.9%) were also purchased from Alfa Aesar, USA. RPMI-1640 medium, phosphate-buffered saline (PBS), tryptic soy broth (TSB), and tryptic soy agar (TSA) were obtained from Life Science Production, UK. Penicillin–streptomycin, phosphate-buffered saline (PBS), and trypsin–EDTA were purchased from Lonza, Germany. 3-[4,5-dimethylthiazol-2-yl]-2,5-diphenyltetrazolium bromide (MTT, ≥ 98%), 2,2-diphenyl-1-picrylhydrazyl (DPPH, ≥ 99%), 2′,7′–dichlorodihydrofluorescein diacetate (DCFH-DA, ≥ 97%), yeast α-glucosidase (≥ 50 U/mg), p-nitrophenyl-α-D-glucopyranoside (pNPG, ≥ 98%), and α-amylase (≥ 2 U/mg) were all supplied by SERVA Electrophoresis GmbH, Heidelberg, Germany. Human lung carcinoma (A549) cell lines were obtained from the Egyptian holding company for biological products and vaccines (Vacsera ), Giza, Egypt.

### Preparation of plant extract

Fresh leaves of *Medicago sativa* L. (alfalfa), *Euphorbia milii* Des Moul. (crown of thorns), *Codiaeum variegatum* (L.) A. Juss. (croton), and *Helianthus annuus* L*.* (sunflower) were purchased from local market, Qena, Egypt, during 14–19 May 2024. The plant species were authenticated by Dr. Mohamed Owis Badry, associate professor in Plant Systematics at Department of Botany & Microbiology, Faculty of Science, South Valley University, Qena (83,523), Egypt. Voucher specimens were prepared and deposited at the South Valley University Herbarium. The identification was conducted according to Boulos (2002, 2009)^14,15^ and updated using the Plants of the World Online database (POWO 2023)^16^. The collected leaves were thoroughly washed with tap water followed by distilled water to eliminate dust and surface impurities. The cleaned leaves were air-dried at room temperature and cut into small fragments. For each plant, 50 g of leaf material were mixed with 500 mL of deionized water and heated at 80 °C on a magnetic heater-stirrer for 30 min. After cooling to room temperature, the mixtures were filtered three times through Whatman No. 1 filter paper. The resulting extracts were stored at 4 °C and later used as reducing and stabilizing agents in the green synthesis of nickel oxide NPs.

### Preparation of green NiO NPs

**Fig. 1:**
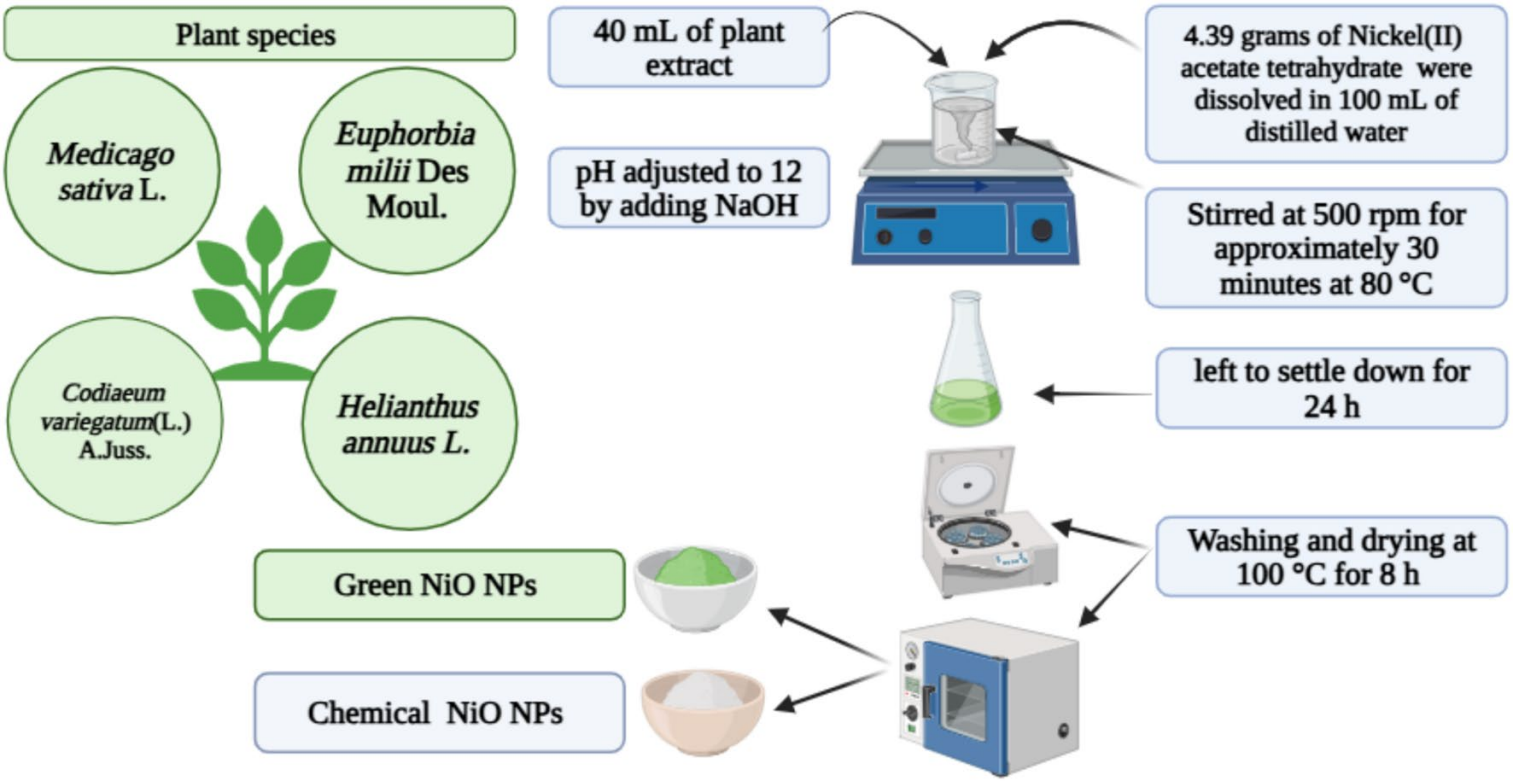
Schematic overview of the green and chemical synthesis of NiO NPs using four plant species. *Medicago sativa* L. (alfalfa), *Euphorbia milii* Des Moul. (crown of thorns), *Codiaeum variegatum A. Juss.* (L.) (croton), and *Helianthus annuus* L*.* (sunflower).

For the synthesis of NiO NPs coprecipitation method was used^17^. Briefly, 4.975 g of nickel acetate were dissolved in 100 mL of distilled water, and the solution was stirred at 500 rpm for approximately 30 min at 80 °C. During this stirring period, 40 mL of plant extract, serving as a reducing and stabilizing agent, was gradually added to the solution. Subsequently, sodium hydroxide (NaOH) was added dropwise to adjust the pH to 12, facilitating the precipitation of NPs. The mixture was then continuously stirred for an additional 2 h to ensure complete precipitation. The resulting precipitate was collected by centrifugation at 10,000 rpm for 10 min at 25 °C and washed several times with a 5:1 mixture of distilled water and ethanol to remove impurities. Schematic illustration of synthesis of NiO NPs via chemical and green routes is shown in Fig. 1. Finally, the purified precipitate was dried at 100 °C for further characterization and biological evaluation.

### Characterization of NiO NPs

X-ray diffraction (XRD) analysis was conducted using an X’Pert PRO PANalytical diffractometer operated at an accelerating voltage of 40 kV and a current of 30 mA. Cu-Kα radiation with a wavelength of 1.54056 Å was employed as the X-ray source. The diffraction patterns were collected to determine the crystalline structure and phase purity of the synthesized NPs. The functional groups present in the samples were examined using Fourier Transform Infrared (FTIR) spectroscopy. Measurements were performed using the KBr pellet method with a Jasco Model 6100 spectrometer (Japan), operating at a resolution of 4.0 cm^-1^ over a wavenumber range of 4000–400 cm^-1^. The optical properties of the samples were evaluated using a UV–visible spectrophotometer (SPECORD 200 PLUS, Analytik Jena, Germany). Absorption spectra were recorded in the range of 200–800 nm to investigate the optical bandgap and electronic transitions. The morphology, particle size, and surface characteristics of the NPs were analyzed using high-resolution transmission electron microscopy (HRTEM). Imaging was performed with a JEOL JEM-2100 microscope (Japan), operated at an accelerating voltage of 200 kV.

### Bioctivity of green NiO NPs

#### MTT assay

Human lung carcinoma (A549) cell lines were seeded in A 96-well tissue culture plate with 1 × 10^5^ cells/mL (100 µl/well) and cultured at 37 °C and 5% CO_2_ for 24 h to form a confluent monolayer. Once the monolayer was established, the growth medium was removed, and the cells were gently washed twice with sterile phosphate-buffered saline (PBS). A stock solution of NiO NPs was prepared at a concentration of 10 mg/mL in sterile PBS, then sonicated for 30 min to ensure uniform dispersion. From this stock, serial dilutions were prepared in RPMI-1640 medium supplemented with 2% fetal bovine serum (maintenance medium) to achieve final working concentrations of 31.25, 62.5, 125, 250, 500, and 1000 µg/mL. A volume of 100 µL of each test sample was added to the respective wells, with three wells designated as controls, receiving only the maintenance medium, while doxorubicin was used as a positive control. An MTT solution (5 mg/ml) was prepared in PBS, and 20 µl was added to each well. The plate was then placed on a shaker and rotated at 150 rpm for 5 min to ensure proper mixing of MTT in the media. The plate was incubated at 37 °C with 5% CO_2_ to allow cellular metabolism of MTT. After incubation, the media was removed, and the plate was rinsed to eliminate any residual material. The formazan product was then solubilized in 200 µL of Dimethyl sulfoxide (DMSO), and the plate was placed on a shaker set at 150 rpm for 5 min to ensure complete dissolution. The optical density was measured at 560 nm, subtracting the background at 620 nm. The amount of formazan is directly proportional to cell viability. All experiments were performed in triplicate, and the average results are presented as a percentage relative to the control. The percentage of cell viability was determined using (Eq. 1)^18^:

1 $$Cell viability(\%) =\frac{\text{ OD of untreated cells}-OD of treated cells}{OD of untreated cells}$$

#### Antioxidant DPPH Assay

The DPPH (2,2-diphenyl-1-picrylhydrazyl) assay is used to assess the antioxidant activity of Ni ONPs. DPPH is a stable free radical characterized by its purple color, and it acts as an efficient radical scavenger. When an antioxidant molecule is introduced, it reduces the DPPH radical, causing the solution to become colorless. In this assay, ascorbic acid is used as positive control. A DPPH stock solution (0.1 mM) was freshly prepared by dissolving 3.94 mg of DPPH in 100 mL of methanol and stored in a dark container to prevent light-induced degradation. In parallel, a stock solution of NiO NPs was prepared at a concentration of 1 mg/mL in methanol and sonicated for 30 min to ensure uniform dispersion. From this stock, serial dilutions were prepared to obtain final working concentrations of 500, 250, 125, 62.5, and 31.25 µg/mL.

For the assay, 3 mL of DPPH solution is added to each test tube containing the prepared dilutions. The tubes are then incubated in the dark at room temperature for 30 min to allow the reaction to proceed. After the incubation, the absorbance (A) of both the control and samples is measured spectrophotometrically at 517 nm. The free radical scavenging activity of the samples is calculated as a percentage using (Eq. 2) (^19^:

2 $$\text{Free radical scavenging activity}\left({\%}\right)= \left(\frac{{\text{Abs}}_{\text{control}}-{\text{Abs}}_{\text{sample}} }{{\text{Abs}}_{\text{control}} }\right)\times 100$$

The antioxidant activity of the extract is quantitatively analyzed by plotting the inhibition percentage against the concentration of the extract. The IC_50_ value, which represents the minimum concentration of the antioxidant required to neutralize 50% of the original DPPH radical, is determined from this graph. This value is a key indicator of the potency of antioxidant material.

#### Antibacterial activity

Minimum inhibitory concentration (MIC) and minimum bactericidal concentration (MBC) were determined to evaluate the antibacterial efficacy of NiO NPs against four bacterial strains: *Staphylococcus aureus* (BLSVU9), *Streptococcus pyogenes* (BLSVU11), *Escherichia coli* (BLSUV1), and *Klebsiella pneumoniae* (BLSVU2). The four bacterial strains were obtained from Bacteriology laboratory, Department of Botany and Microbiology, Faculty of Science, South Valley University, Qena, Egypt.

A stock solution of NiO NPs was prepared at 10 mg/mL in sterile distilled water and sonicated for 30 min to ensure homogeneous dispersion. Serial two-fold dilutions were then prepared in sterile tryptic soy broth (TSB) to obtain the desired range of concentrations for the MIC assay. The minimum inhibitory concentration (MIC) was determined by microbroth dilution using p-iodonitrotetrazolium violet chloride (INT) as an indicator. Overnight bacterial cultures grown on tryptic soy agar (TSA) were suspended in TSB and adjusted to an optical density (OD_600_) of 0.001. A 100 µL aliquot of this bacterial suspension was added to each well of a 96-well microtiter plate, followed by 100 µL of NiO NPs solutions at various concentrations (prepared via serial dilution from the stock). TSB medium with and without tested bacteria were served as two controls. Plates were then incubated at 37 °C for 24 h. After incubation, 40 µL of INT solution (0.2 mg/mL) was added to each well, followed by an additional 2 h incubation at 37 °C^20,21^. The MIC was defined as the lowest concentration of the test sample that prevented any visible color change, indicating the absence of bacterial growth^22^.

MBC was determined by transferring 50 µL from each non-turbid well of the MIC plate to fresh, TSA plates. After 24 h of incubation at 37 °C, viable bacterial colonies were counted. The MBC was defined as the lowest concentration of the extract that resulted in a 99.9% reduction in colony-forming units (CFU) compared to the initial inoculum (a 3-log reduction). The detection limit for this assay was 10^1^ CFU/mL^23^.

#### Antibiofilm activity

The ability of target bacterial strains to form static biofilms was initially evaluated using the crystal violet staining assay in 96-well microtiter plates^24,25^. A stock solution of NiO NPs was prepared at a concentration of 10 mg/mL in sterile distilled water, followed by sonication for 30 min to ensure uniform dispersion. To evaluate biofilm formation, Overnight Bacterial culture suspended in TSB were adjusted to an OD_595_ of 0.02. Then, 130 µL of each diluted isolate was transferred into the wells of a U-bottom 96-well microtiter plate (Sterilin) and incubated at 37 °C for 24 h to allow biofilm formation. Following incubation, the wells were gently washed with distilled water to remove non-adherent cells. The remaining biofilm was stained with 0.1% crystal violet for 10 min (170 µL). Excess stains were removed by washing the wells with distilled water, and the bound dye was solubilized using 210 µL of 96% ethanol. The absorbance was then measured at 595 nm using an Infinite® F50 Robotic microplate reader (Austria) to quantify the amount of biofilm formed^26^. For the biofilm inhibition assay, 30 µL of the tested extracts were added to each well of previously incubated plates, followed by a second 24 h incubation. After incubation he wells were washed and stained as described previously to measure the residual biofilm mass^27^.

#### Anti-inflammatory

A cyclooxygenase (COX-1) inhibition assay was conducted to assess the anti-inflammatory activity of the tested samples. A stock solution for each test sample was prepared by dissolving 10 mg of NiO NPs in 1 mL of DMSO (10 mg/mL). This solution was then serially diluted with DMSO to obtain working concentrations ranging from 0.5 to 1000 μg/mL and pre-incubated with the COX-1 enzyme at 25 °C for 5 min in the presence of hematin (1 μM). The reaction mixture included phenol (500 μM), 1-leuco-dichlorofluorescein (20 μM), and arachidonic acid (50 μM), all dissolved in 0.1 M Tris buffer (pH 8.0). A blank sample was prepared without the enzyme to serve as a control. Celecoxib was used as the standard. Following the addition of the substrate mix, the absorbance was recorded at 502 nm and the percentage of inhibitory was evaluated using (Eq. 3)^28^:

3 $$(COX-1 )Inhibition \% =\left(\frac{{\text{Abs}}_{\text{control}}-{\text{Abs}}_{\text{sample}} }{{\text{Abs}}_{\text{control}} }\right)\times 100$$

The IC_50_ value (μg/mL), representing the concentration required to inhibit 50% of COX-1 activity, was calculated from the inhibition curve.

#### Antidiabetic assay

The inhibitory effects of the synthesized samples on key carbohydrate hydrolyzing enzymes, α-amylase and α-glucosidase, were evaluated to assess their potential antidiabetic activity. The α-amylase inhibition assay was conducted using the 3,5-dinitrosalicylic acid (DNSA) method. A stock solution of each NiO NPs sample was prepared by dissolving 10 mg of the sample in 1 mL of 10% DMSO (10 mg/mL). Working concentrations ranging from 1.9 to 1000 μg/mL were prepared by serial dilution using phosphate buffer (0.02 M Na₂HPO₄/NaH₂PO₄ and 0.006 M NaCl, pH 6.9). In this assay, 200 μL of α-amylase (2 U/mL) was mixed with an equal volume of the extract and incubated at 30 °C for 10 min. Then, 200 μL of 1% starch solution was added, and the reaction continued for 3 min. To terminate the enzymatic activity, 200 μL of DNSA reagent was added, and the mixture was heated in a water bath at 85–90 °C for 10 min, cooled, and diluted with 5 mL distilled water. The absorbance was recorded at 540 nm using a UV–Visible Biosystem 310 spectrophotometer. A control without extract (100% enzyme activity) and blank samples without enzyme were used for comparison. The percentage of α-amylase inhibition was calculated using (Eq. 4)^29^:

4 $$\alpha -\text{amylase }inhibition (\%) =\frac{{\text{A}}_{\text{Control}}- {\text{A}}_{\text{Sample}}}{{\text{A}}_{\text{Control}}} \times 100$$

For the α-glucosidase inhibition assay, yeast α-glucosidase and p-nitrophenyl-α-D-glucopyranoside (pNPG) were employed. A stock solution of NiO NPs (20 mg/mL) was prepared in 10% DMSO and further diluted to obtain final working concentrations ranging from 0.5 to 5.0 mg/mL. Test samples and Acarbose (positive control) were mixed (100 μL of 2–20 mg/mL) with 50 μL of α-glucosidase (1 U/mL) and 250 μL of 0.1 M phosphate buffer (pH 6.9). After pre-incubating at 37 °C for 20 min, 10 μL of 10 mM pNPG was added to each mixture, followed by another incubation for 30 min. The reaction was halted by adding 650 μL of 1 M sodium carbonate, and the absorbance was measured at 405 nm using an Amersham Biosciences spectrophotometer. The α-glucosidase inhibition percentage was calculated using (Eq. 5)^30^:

5 $$\alpha -glucosidase inhibition (\%) =\frac{{\text{A}}_{Control}- {\text{A}}_{\text{Sample}}}{{\text{A}}_{Control}} \times 100$$

wherein A_Control_ is the absorbance of the control without protein extract, and A_Sample_ is the absorbance of sample containing the protein extract. In both assays, inhibition percentages were plotted against concentrations to estimate the IC_50_ values, representing the concentration required to inhibit 50% of the enzyme activity.

### Statistical analysis

Statistical variation among the obtained results was assessed using one-way analysis of variance (ANOVA) with the Statistical Package for the Social Sciences. Data are presented as the mean ± standard deviation (SD). Differences were considered statistically significant at P < 0.05.

## Results and discussion

### Mechanism of bioreduction of NiO NPs

**Fig. 2:**
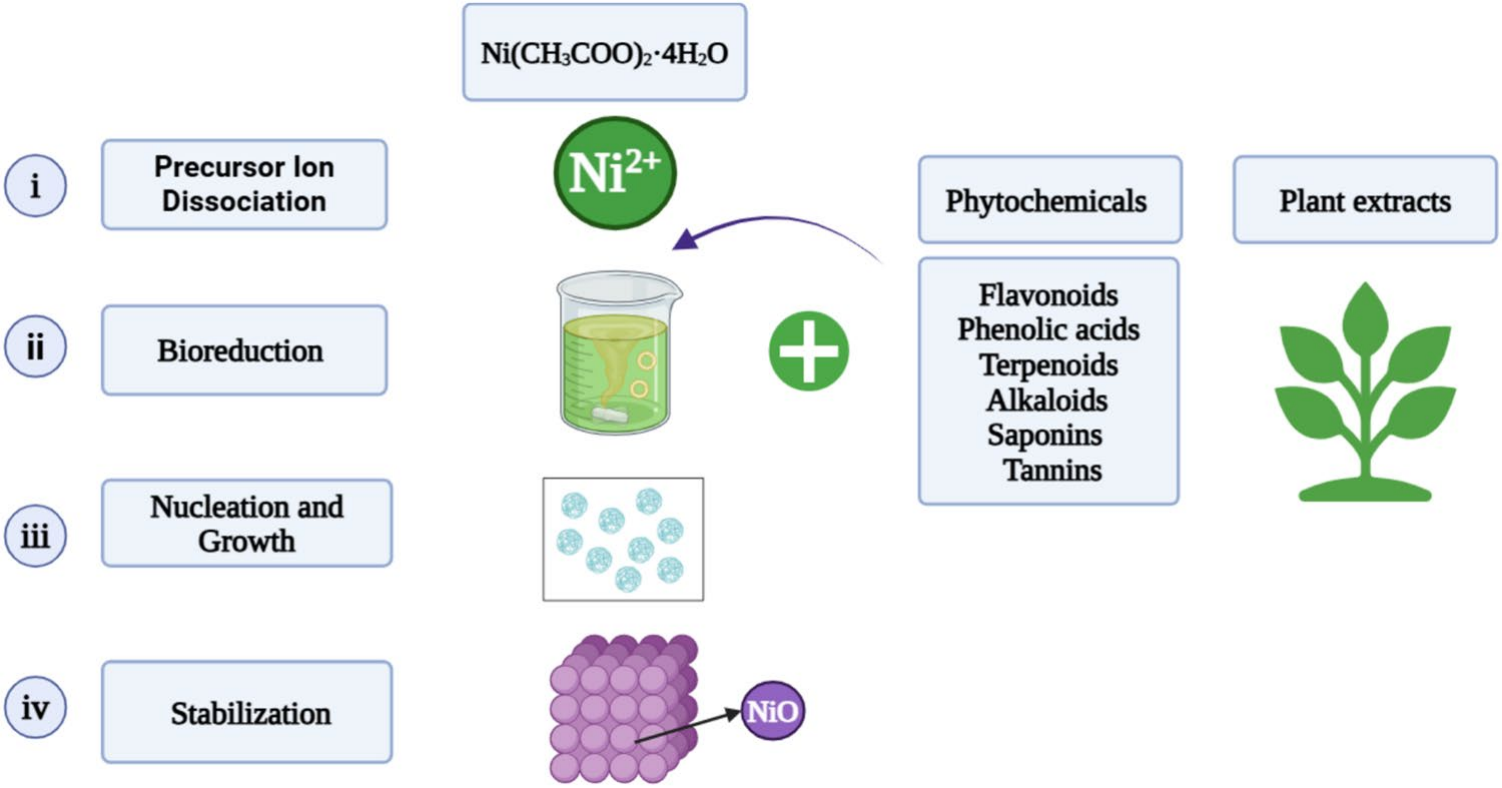
Illustration of bioreduction mechanism utilized in green synthesis of NiO NPs.

The bioreduction mechanism used in the green synthesis of NiO NPs uses phytochemicals derived from plant extracts as organic stabilizing, capping, and reducing agents as illustrated in Fig. 2.

Following the addition of nickel acetate tetrahydrate (Ni(CH_3_COO)_2_·4H_2_O) to an aqueous plant extract, the following stages take place^31^:

(i) Precursor ion dissociation.

In aqueous solution, nickel acetate dissociates into Ni^2+^ and acetate ions:

$${\text{Ni}}\left( {{\text{CH}}_{3} {\text{COO}}} \right)_{2} \cdot 4{\text{H}}_{2} {\text{O }} \to {\text{ Ni}}^{2 + } + \, 2{\text{CH}}3{\text{COO}}^{ - } + 4{\text{H}}_{2} {\text{O}}$$

(ii) Bioreduction.

Plnt extracts contain phytochemicals that can donate electrons, including flavonoids, phenolics, tannins, saponins, terpenoids, and alkaloids. These phytochemicals have hydroxyl (OH^−^), carboxyl (COOH^−^), and aldehyde (CHO^−^) groups. Ni^2+^ ions are reduced to nickel oxide by these groups:

$${\text{Ni}}^{2 + } + {\text{Reductive}}\;{\text{phytochemicals}} \to {\text{NiO NPs}} + {\text{Oxidized}}\;{\text{ phytochemicals}}.$$

(iii) Nucleation and growth.

Nuclei of reduced NiO start to develop and expand. The size and form are determined by the type and availability of the phytochemicals, and the process continues as long as reducing agents are present^32^.

(iv) Stabilization.

By capping, phytochemicals that reduce Ni^2+^ stabilize the surface of NiO NPs and prevent agglomeration. This enhances colloidal stability and results in a more consistent particle distribution^32^.

### Characterization of green NiO NPs

#### X‑ray Diffraction (XRD)

**Fig. 3:**
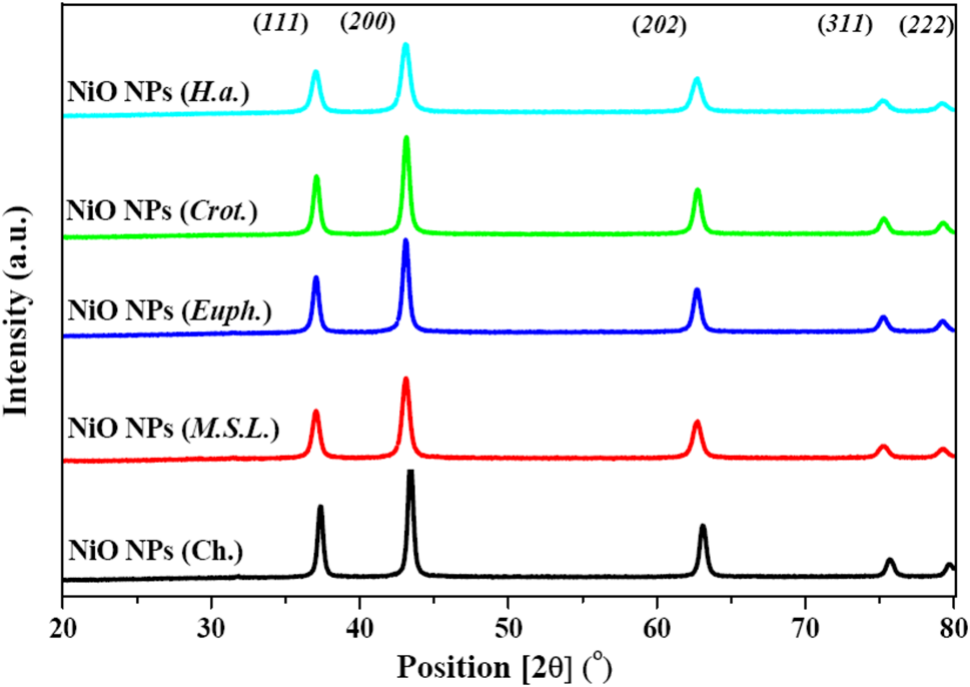
XRD patterns of NiO NPs synthesized via chemical and green routes.

The X-ray diffraction (XRD) patterns of both green and chemically synthesized NiO NPs are presented in Fig. 3. All five samples exhibited well-defined diffraction peaks at 2θ values of approximately 37.2°, 43.2°, 62.8°, 75.3°, and 79.3°, which correspond to the (111), (200), (220), (311), and (222) crystallographic planes, respectively. These peaks are consistent with the standard face-centered cubic (FCC) crystal structure of NiO NPs and match well with the reference database (COD) card no. 4320490 and JCPDS card no. 47–1049, confirming the successful formation of single-phase NiO NPs^12^.

The lack of secondary peaks suggests that there are no contaminants or secondary phases present. The basic crystalline structure remained unchanged when various plant extracts were used. Nevertheless, there were differences in peak intensity between the samples. Indicating a higher degree of crystallinity, the chemically synthesized NiO (Ch.) NPs had the maximum luminosity at 2θ = 43.2° (corresponding to the (200) plane). The increased thermal energy supplied during chemical synthesis, which improves lattice atom mobility and fosters better crystal development, could be the cause of this rise^33^. Among the green-synthesized NPs, those prepared using *Euphorbia milii Des Moul. milii* and *Codiaeum variegatum* (L.) A. Juss. extracts showed relatively stronger peak intensities, indicating moderate crystallinity. In contrast, the sample synthesized with *Helianthus annuus* L*.* extract exhibited the lowest peak intensity, suggesting a lower degree of crystallinity in that particular sample.

The interplanar spacing (d) was calculated using Bragg’s law, and the Miller indices (*h k l*) for the NiO NPs were assigned based on the standard diffraction data corresponding to COD card number 4320490. The following equation was used for the d-spacing calculation (Eq. 6)^34^:

6 $$n\lambda = 2d \, \sin \, \theta$$

where *n* is the order of diffraction, *λ* is the wavelength of the incident X-ray (1.5406 Å), *d* is the interplanar spacing (in Å), and *θ* is the diffraction angle (in radians).

Since NiO possesses a cubic crystal structure, the lattice constants are equal (*a* = *b* = *c* = 4.18720 Å). The lattice parameter of the green-synthesized NiO NPs was calculated using the following (Eq. 7)^35^:

7 $$\frac{1}{{d}^{2}} = \frac{{h}^{2}+{k}^{2}}{{a}^{2}}+\frac{{l}^{2}}{{c}^{2}}$$

where *d* is expressed in angstroms (Å), *h, k,* and *l* are the Miller indices, and *a,b* and *c* represent the lattice parameters of the crystal structure. To estimate the average crystallite size of the NiO NPs, the Scherrer equation was applied (Eq. 8)^36^,

8 $$D=\frac{K\lambda }{\beta \mathit{cos}\theta }$$

where D is the crystal size, K is the shape factor (typically ~ 0.9), λ is the wavelength of the X-ray, θ is the Bragg angle, and β is the observed peak width at half-maximum height.

The crystallite sizes of the NiO NPs were found to vary between 18.614 nm and 29.329 nm, indicating the successful synthesis of nanoscale materials across all samples are illustrated in Table 1. Among the different samples, NiO (Ch.) NPs exhibited the largest average crystallite size (29.329 ± 8.78 nm), while NiO (*M.S.L.*) NPs displayed the smallest size (18.614 ± 2.74 nm). The variations in particle size can be attributed to the nature and concentration of phytochemicals present in the plant extracts used during the synthesis process, which influence nucleation and growth rates during NPs synthesis^37^. This is consistent with findings from other research that use green synthesis: The crystallite diameters of NiO NPs synthesized using *Syzygium cumini* extract were roughly 23 nm^38^, whereas those made with plant-mediated syntheses that included *Catharanthus roseus* and *Azadirachta indica* were between 22 and 28 nm^39^. Moreover, crystallites of about 21 nm in size were produced by synthesis using *Moringa oleifera*, and biosynthesized NiO particles made with *Allium cepa* stalks extract measured between 18 and 25 nm^7,40^.

**Table 1:** Crystal structure parameters of the synthesized NiO NPs s extracted from XRD data.

Sample	Crystal size (nm)	Lattice constant (*a* = *b* = *c*) (Å)	Unit cell volume (Å)^3^
NiO (*M.S.L.*) NPs^1^	18.614 ± 2.74	4.19112 ± 0.0042	73.62 ± 0.22
NiO (*Euph.*) NPs^2^	20.403 ± 2.23	4.192072 ± 0.005	73.67 ± 0.28
NiO (*Crot.*) NPs^3^	24.424 ± 5.17	4.192636 ± 0.005	73.79 ± 0.27
NiO (*H.a.*) NPs^4^	21.511 ± 2.87	4.190184 ± 0.002	73.57 ± 0.15
NiO (Ch.) NPs^5^	29.329 ± 8.78	4.17626 ± 0.002	72.83 ± 0.12

^1^*Medicago sativa* L*.*, ^2^ *Euphorbia milii* Des Moul., ^3^ *Codiaeum variegatum* (L.) A. Juss., ^4^ *Helianthus annuus* L*.* and ^5^ Chemically synthesized sample.

The calculated lattice constants (*a* = *b* = *c*) for the NiO NPs samples were close to the standard value for bulk cubic NiO, with slight variations observed. NiO (*Crot.*) NPs exhibited the highest lattice constant (4.192636 ± 0.005 Å), whereas NiO (Ch.) NPs showed a slightly reduced lattice constant (4.17626 ± 0.002 Å). These minor deviations suggest the presence of microstrain or slight distortions in the crystal structure, possibly arising from the incorporation of plant-derived organic residues or differences in synthesis conditions^41^. Correspondingly, the unit cell volumes ranged from 72.83 Å^3^ for NiO (Ch.) NPs to 73.79 Å^3^ for NiO (*Crot.*) NPs. The observed increase in unit cell volume for certain samples, particularly NiO (*Crot.*) NPs aligns with the increase in lattice constant and suggests a slight expansion of the crystal lattice. This expansion may be due to the incorporation of defects or surface functional groups originating from the phytochemical components during the green synthesis.

Overall, the XRD results, combined with crystallographic parameters, strongly confirm the formation of highly crystalline, cubic-phase NiO NPs. The subtle variations in size and lattice parameters among the different NiO NPs reflect the significant role of green synthesis conditions on the structural properties. These structural characteristics are expected to have a direct impact on the biological and catalytic activities of NiO NPs, as observed in subsequent cytotoxicity and antioxidant studies.

#### High Resolution Transmission Electron Microscope (TEM)

**Fig. 4:**
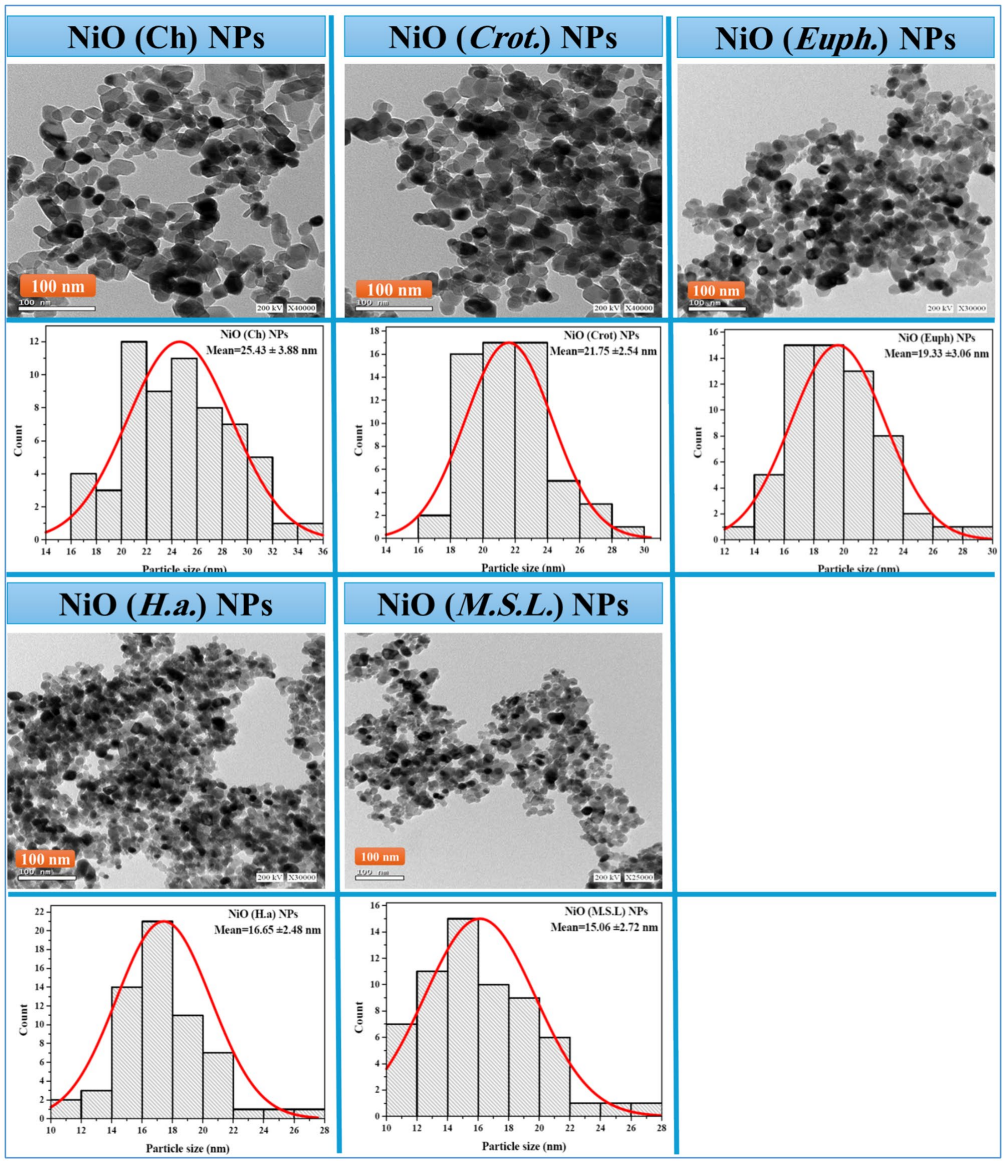
HRTEM image and size distribution histogram of NiO NPs. Images with scalebar 100 nm were recorded at 200 kV with magnification X40000. Particle size distribution was calculated with image j software (n = 100).

The morphological characteristics and particle size distributions of NiO NPs were investigated using high-resolution transmission electron microscopy, as shown in Fig. 4. The observed particle sizes ranged from 15 to 25 nm, in good agreement with the crystallite sizes calculated from X-ray diffraction data using Scherrer’s equation. This consistency between microscopic and crystallographic analyses confirms the effectiveness and reliability of the employed synthesis protocols in producing nanocrystals with well-defined dimensions.

The chemically synthesized NiO NPs exhibited a mean particle size of 25.43 ± 3.88 nm and showed pronounced agglomeration, with a quasi-spherical to irregular morphology. This aggregation is likely due to the absence of capping agents, which play a critical role in preventing particle–particle fusion during the nucleation and growth stages^42^. However, beyond the lack of stabilizers, agglomeration can also be attributed to the high surface energy of NPs in aqueous suspension, which enhances van der Waals attractions. Furthermore, hydrogen bonding interactions, especially in the presence of surface hydroxyl groups, can promote clustering, as discussed in recent studies such as during the synthesis of copper oxide NPs using *Colebrookea oppositifolia*^43^.

Green-synthesized NiO NPs mediated by *Medicago sativa* L. extract exhibited the smallest average particle size of 15.06 ± 2.72 nm, with spherical morphology and minimal agglomeration. The improved morphological control is attributed to the rich content of bioactive compounds such as flavonoids, phenolic acids, and saponins in the *Medicago sativa* extract. These phytochemicals function as both reducing and stabilizing agents, enabling uniform nucleation and limiting uncontrolled growth, thereby producing smaller and more monodisperse NPs^44^.

NiO NPs synthesized using *Euphorbia milii* Des Moul. extract showed a mean particle size of 19.33 ± 3.06 nm with moderate aggregation. This sample predominantly displayed spherical morphology. The plant’s extract contains tannins, terpenoids, and alkaloids, which possess known reducing and stabilizing capabilities. However, their effectiveness in providing steric or electrostatic stabilization appears to be less than that observed for *Medicago sativa* L., resulting in moderate control over nanoparticle size and shape^7^.

In the case of *Croton*-mediated NiO NPs, the mean particle size was 21.75 ± 2.54 nm, with extensive aggregation and broader size distribution. Although this plant contains bioactive compounds such as diterpenoids and phenolics, their ability to serve as effective capping agents may be limited due to lower concentration or weaker surface-binding affinity. As a result, less regulated particle growth leads to polydisperse and aggregated structures^37^.

NiO NPs synthesized using *Helianthus annuus* L*.* extract had an average size of 16.65 ± 2.48 nm and demonstrated relatively uniform morphology with acceptable dispersion. The effective size control and reduced aggregation in this sample are attributed to the presence of flavonoids, chlorogenic acid, and other polyphenolic compounds in the sunflower extract, which contribute to the reduction of nickel ions and help stabilize the NPs surfaces^17^.

According to a comparison of particle sizes and morphologies, the smallest and best dispersed NiO NPs are produced by green synthesis employing *M. sativa* and *H. annuus*. This is explained by their individual phytoconstituents’ potent reducing and capping properties. On the other hand, the chemically produced and *Croton* mediated NiO NPs showed higher levels of aggregation and larger particle sizes, underscoring the drawbacks of plant extracts with less capping potential and non-biogenic synthesis.

#### Fourier transformation Infrared spectroscopy (FTIR)

**Fig. 5:**
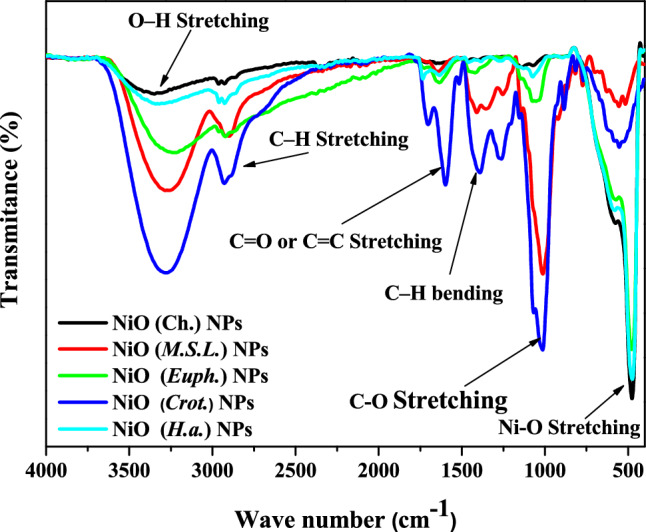
FTIR spectra of NiO NPs synthesized via chemical and green routes.

The FTIR spectra of NiO NPs synthesized via both chemical and green routes are presented in Fig. 5. All samples showed a noticeable absorption band between 430 and 600 cm^−1^, which corresponds to the Ni–O stretching vibration and confirming the successful formation of NiO as well as the presence of surface-bound functional groups. This band’s presence across all spectra suggests that the metal oxide framework was maintained irrespective of the synthesis method^31,40^. A broad absorption band centered around 3400–3800 cm^−1^, particularly prominent in green-synthesized NiO NPs, is associated with O–H stretching vibrations from hydroxyl groups or adsorbed moisture. This increase in intensity in plant-derived samples reflects a higher content of hydrophilic surface functionalities, likely from phytochemicals such as flavonoids, polyphenols, and phenolic acids^12^. A medium-intensity band in the 1620–1660 cm^−1^ range, present in all samples, is attributed to O–H bending or C = O stretching modes, further indicating water adsorption or the presence of carbonyl compounds^38^.

The weak band observed at ~ 2900 cm^−1^ corresponds to aliphatic C–H stretching vibrations. This feature, more noticeable in green-synthesized samples, confirms the incorporation of organic residues from plant extracts during the synthesis. Broad and overlapping peaks between 1360–1800 cm^−1^ reflect various C = O and C = C stretching vibrations, indicating the presence of organic compounds such as tannins and terpenoids, which may act as capping or stabilizing agents in the green synthesis route. Additional peaks in the ~ 1030–1100 cm^−1^ region, which are indicative of C–O stretching vibrations linked to alcohols, ethers, and polysaccharides, were also visible in the FTIR spectra of the green-synthesized NPs^9,45^. These peaks support the plant-based chemicals’ capping and potential stabilizing function on the surface of the NPs.

The *Medicago sativa*-derived NiONPs showed sharper peaks and moderate intensity, especially at ~ 3400 cm^−1^ and ~ 1630 cm^−1^. This suggests that phenolic O–H and amide groups are present in more homogeneous binding contexts or at relatively lower quantities^3^. On the other hand, the broad and intense O–H and C = O bands observed in *Euphorbia milii*-mediated NiO were suggestive of a high concentration of hydrogen-bonded hydroxyl and carbonyl groups, which is in line with the plant’s rich polyphenolic and triterpenoid profile^39^. A large O–H band and a comparatively strong aliphatic C-H stretching near 2900 cm^−1^ were seen in the *Helianthus annuus*-synthesized NiO NPs, which might be ascribed to lipidic chemicals and long-chain biomolecules. This suggests that the reduction and capping processes involve a wide range of phytochemicals^46^. Curiously, the *Codiaeum variegatum* NiO NPs showed the broadest and most intense bands in all main regions, particularly in the 1000–1400 cm^−1^ range, suggesting a complex mixture of amino-functional chemicals, alcohols, and esters. Significant peak broadening and overlap indicate a higher degree of surface functionalization and numerous binding contexts, which could improve the particle’s colloidal stability and reactivity^47^.

These spectral features are consistent with prior studies on green-synthesized NiO NPs^48,49^, and highlight the stabilizing role of bio-organic functional groups in influencing nanoparticle morphology, dispersion, and potential application behavior A detailed summary of the functional group assignments and their corresponding peak positions is presented in Table 2.

**Table 2:** Functional group assignments identified in the FTIR spectra of NiO NPs.

FTIR peak assignment table with references				
Wavenumber (cm^-1^)	Observed In	Assignment	Possible Origin	References
~ 3300–3400	All samples	O–H stretching (broad)	Adsorbed water, hydroxyls from alcohols, phenols	^12^
~ 2900–2920	Green NPs	C–H stretching (asymmetric/symmetric)	Aliphatic –CH_2_/–CH_3_ groups from plant metabolites	^9,45^
~ 1630–1650	All samples	H–O–H bending / C = C or amide I stretching	Water molecules, aromatic rings, amides from proteins	^38^
~ 1380–1410	Green NPs	C–N stretching / COO⁻ symmetric stretch	Amino acids, polyphenols, or proteins	^48,49^
~ 1230–1280	Green NPs	C–O–C or C–N stretching	Esters, ethers, or amines from plant biomolecules	^48,49^
~ 1030–1100	Green NPs	C–O stretching / C–OH bending	Carbohydrates, polyols, or polysaccharides	^9,45^
~ 500–600	All samples	Ni–O stretching vibration	Nickel–oxygen lattice vibrations (NiO confirmation)	^17^

#### UV–Vis Spectroscopy

**Fig. 6:**
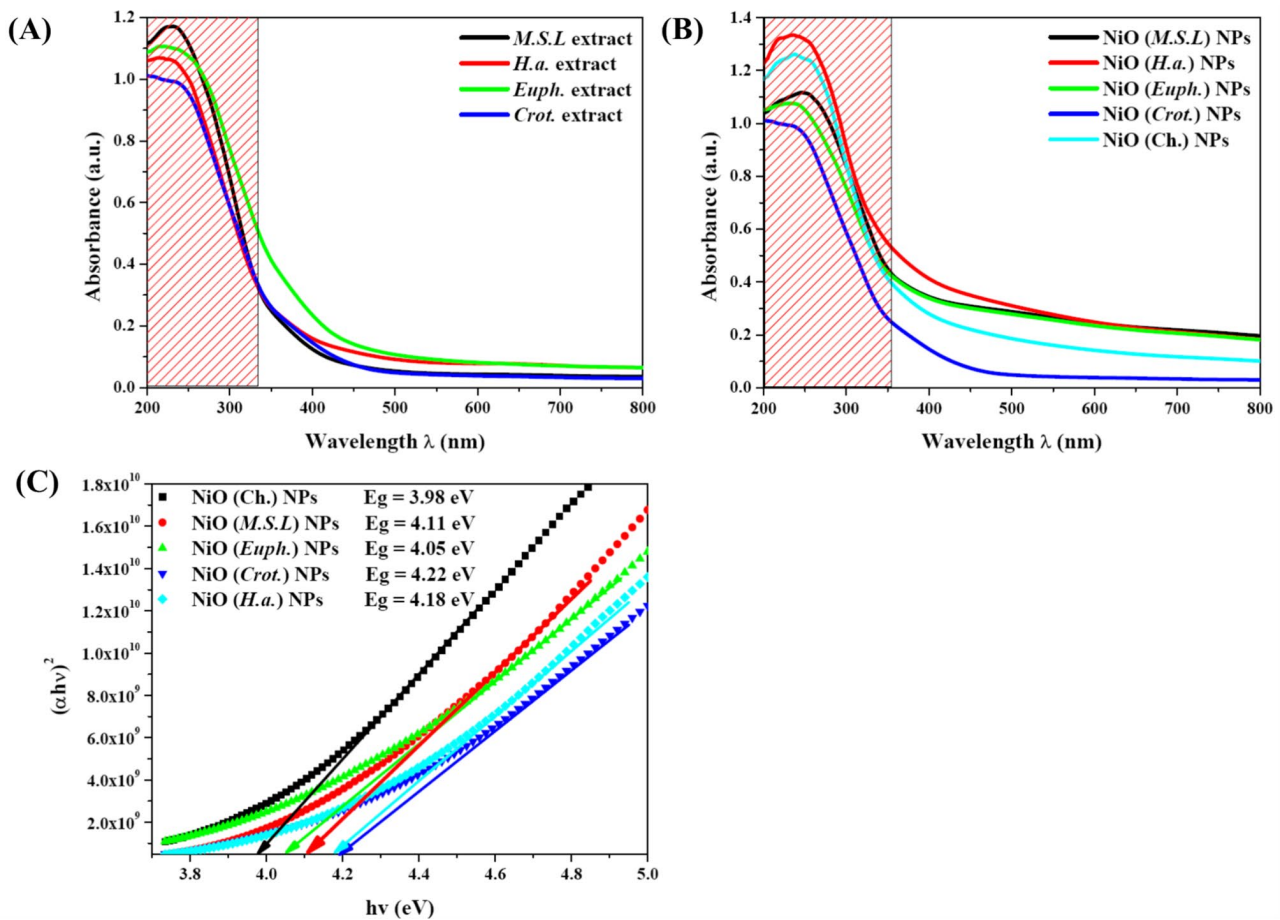
Optical properties of NiO NPs: UV–Vis spectra of the plants extract (**A**), UV–Vis spectra of NiO NPs synthesized using chemical and green routes (**B**) and Tauc plot for calculating optical bandgap energies of the synthesized NiO NPs (**C**).

In order to investigate the role of plant extracts on the optical properties of NiO NPs, UV–Vis spectroscopy was performed. The UV–Vis absorption spectra of the selected four plant extracts, *Medicago sativa* L*.*, *Helianthus annuus* L., *Euphorbia milii* Des Moul., and *Codiaeum variegatum* (L.) A. Juss., were recorded as shown in Fig. 6** (A)**. The UV–Vis absorption spectra of all plant extracts showed noticeable peaks in the 270–330 nm region, which are indicative of the π → π* and n → π* transitions linked to aromatic and conjugated phytochemicals such flavonoids, tannins, and polyphenols^50^. These bioactive phytochemicals serve as stabilizing agents that stop NPs agglomeration and as reducing agents for Ni^2+^ ions in the green synthesis process^51,52^.

The UV–Vis spectra of the synthesized NiO NPs revealed strong absorption in the UV region, with absorption edges slightly shifted depending on the used plant extract as shown in Fig. 6** (B)**. Strong absorption bands, mostly in the 280–370 nm range, were observed in the UV–Vis absorption spectra of the produced NiO NPs, which are revealing of intrinsic electronic transitions inside the NiO lattice. According to earlier reports on the behavior of NiO semiconductors, these transitions are specifically ascribed to the charge transfer from O^2−^ (2p) orbitals to Ni^2+^ (3d) orbitals^53^. The absorption edges’ sharpness and location differed according to the synthesis technique and the plant extract used, suggesting that the phytochemical environment during synthesis had a direct impact on the NPs optical response. Significantly, all of the green synthesized NiO samples showed a blue shift when compared to chemically synthesized NiO NPs, which was supported by TEM and XRD results and suggested the formation of smaller NPs. These changes in absorption edges have a direct effect on the materials optical band gaps, suggesting the influence of surface alteration and quantum confinement^17^.

The optical bandgap energy (Eg) of the synthesized NPs was determined according to Tauc’s Eq. (9)^54^:

9 $$\alpha \left( {{\text{h}}v} \right) = {\text{A}}\left( {{\text{h}}v - {\text{E}}_{{\text{g}}} } \right)^{{{\text{m}}/2}}$$

where α is the absorption coefficient, hν is the photon energy, A is a proportionality constant, and m is an exponent that depends on the nature of the electronic transition. For direct allowed transitions, m = 1, whereas for indirect allowed transitions, m = 4. Subsequently, plots of (αhν)^2^ versus hν (photon energy) were constructed, and the optical bandgap was then determined by projecting the linear section of the curves to intercept the energy axis (ℎν hν) at (αℎν)^2^ = 0.

The results showed that the optical bandgap energies of green synthesized NiO NPs ranged from 4.05 eV to 4.22 eV, which is somewhat higher than that of chemically synthesized ones, whereas NiO (Ch.) NPs had values of 3.98 eV, Fig. 6C. The largest bandgap (4.22 eV) among the green synthesized NPs was found in NiO (Crot.) NPs, which may be related to improved stability and stronger surface capping by the phytochemicals found in *Croton* extract^55^. It is well known that these phytochemicals, which are abundant in polyphenols, flavonoids, and tannins, form an organic shell around NPs, causing further electronic confinement and surface state changes^3^.

### Bioctivity of green NiO NPs

According to the obtained physicochemical characterization results, the green synthesized NiO NPs exhibited no significant structural changes, with only slight variations in particle size, ranging from 15 to 21 nm. Therefore, we can emphasize that the variations of the bioactivity of the green NiO NPs are predominantly influenced by the phytochemical composition of the plant extracts used during the synthesis. Table 3 highlights the critical role of plant derived biomolecules, such as flavonoids, terpenoids, phenolic acids, and proteins, in modulating the surface properties, stability, and biological performance of the resulting NPs.

**Table 3:** Summery of specification, phytochemicals, bioactivities of the used plants.

Plant	Plant specification	Major phytochemicals	Main bioactivities	Examples of NPs synthesized	[R]
*Medicago sativa* L. (Alfalfa)	Perennial herbs; leaves, stems, seeds	Flavonoids (apigenin, quercetin), saponins, phytoestrogens, coumarins	Antioxidant, anti-inflammatory, antimicrobial, cardioprotective, estrogenic	Ag NPs, Au NPs, NiO NPs, ZnO NPs	^56–58^
*Euphorbia milii* Des Moul. (Crown of Thorns)	Spiny shrub; latex, leaves	Terpenoids, flavonoids, alkaloids, latex proteins	Antimicrobial, cytotoxic, anti-inflammatory, larvicidal	Ag NPs, ZnO NPs, Fe_3_O_4_ NPs	^59–61^
*Codiaeum variegatum* (L.) A. Juss. (Croton)	Evergreen shrub; colorful leaves	Flavonoids, tannins, alkaloids, terpenoids	Antioxidant, antibacterial, antifungal, cytotoxic	Ag NPs, Au NPs, ZnO NPs,	^62–64^
*Helianthus annuus* L. (Sunflower)	Annual herb; seeds, leaves, flowers	Phenolic acids (chlorogenic acid, caffeic acid), flavonoids, tocopherols	Antioxidant, anti-inflammatory, antimicrobial, wound healing, antidiabetic	Ag NPs, ZrO_2_ NPs, ZnO NPs	^65–67^

#### The mechanism of bioactivity of green NiO NPs

**Fig. 7:**
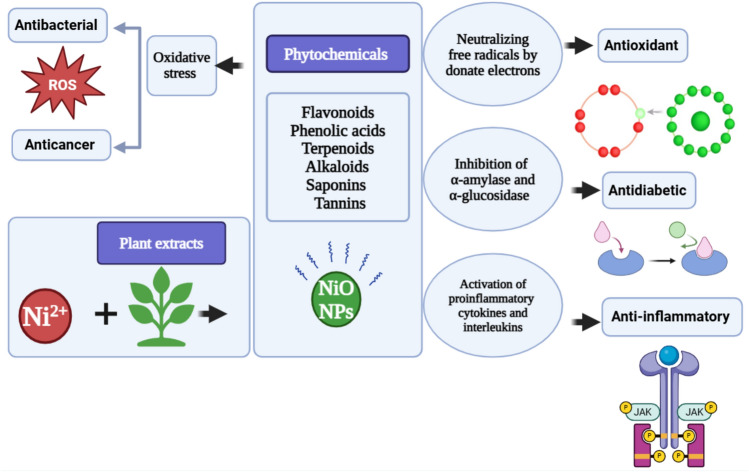
Illustration of the different bioactivities of green synthesized NiO NPs.

Figure 7 illustrates the possible mechanisms of different bioactivates of green NiO NPs. ROS are major contributors to antibacterial and anticancer activity because they cause oxidative stress, which harms the DNA and membranes of bacteria and cancer cells. On the other hand, oxidative stress is neutralized, and free radicals are scavenged by phytochemicals and NiO NPs through electron donation processes, which contribute to the antioxidant activity. While, inhibiting the digestive enzymes α-amylase and α-glucosidase reduces the breakdown and absorption of glucose, which further contributes to the antidiabetic activity. Additionally, modification of inflammatory pathways, including the inhibition of proinflammatory cytokines and interleukins, most likely due to the inhibition of JAK/STAT signaling, results in anti-inflammatory characteristics.

#### Cytotoxicity of green NiO NPs

**Fig. 8:**
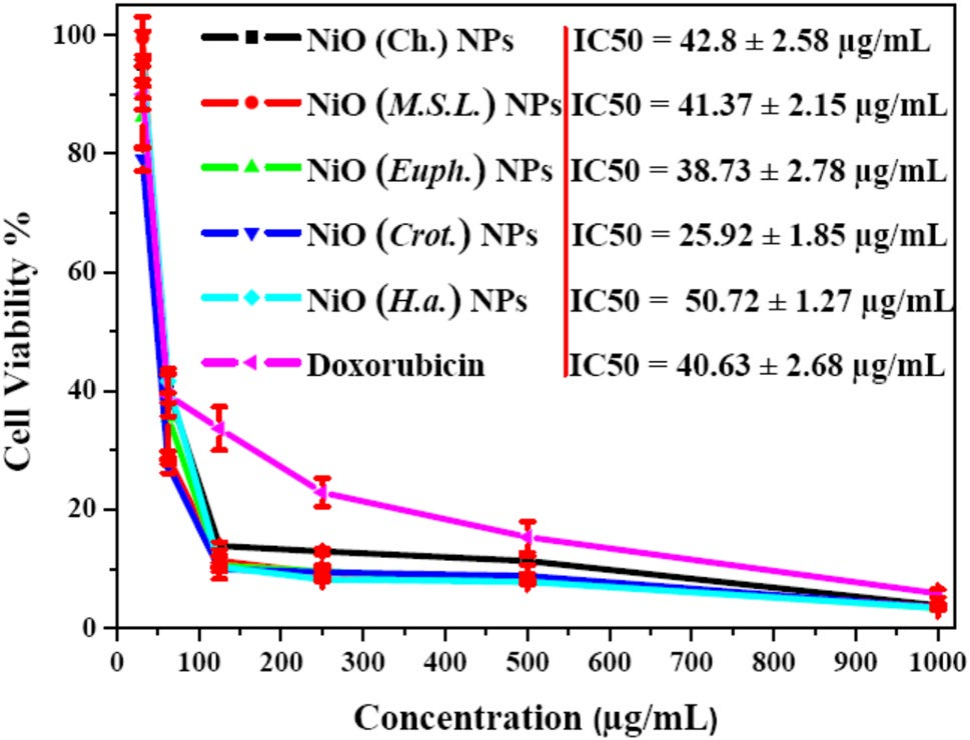
Cytotoxicity of NiO NPs synthesized via chemical and green routes against A549 cell lines at different concentrations (31.5–1000 µg/mL).

The cytotoxic effects of NiO NPs against A549 human lung carcinoma cell lines were evaluated using the MTT assay, as presented in Fig. 8. All tested concentrations (31.5–1000 µg/mL) induced a significant, concentration-independent reduction in cell viability. In comparison to the standard doxorubicin, NiO (*Crot.*) NPs notably demonstrated the highest cytotoxicity, reducing viability to 79.1% at the lowest tested concentration (31.5 µg/mL), followed by NiO (*Euph.*) (86.1%), NiO (Ch.) (95%), NiO (*H.a.*) (95.5%), and NiO (*M.S.L.*) (99.4%) NPs. At the highest concentration (1000 µg/mL), the reduction in cell viability for all samples ranged between 3.41% and 3.85%, suggesting that NiO NPs retained strong anticancer activity even at elevated doses^68,69^.

The half-maximal inhibitory concentration (IC_50_) values further support these observations. Compared with the standard doxorubicin (IC₅₀ = 40.63 ± 2.68 µg/mL), NiO (*Crot.*) NPs recorded the lowest IC₅₀ value at 25.922 ± 1.858 µg/mL, indicating the highest cytotoxic potency. In contrast, NiO (H.a.) NPs exhibited the highest IC_50_ value of 50.726 ± 1.276 µg/mL. This biological activity can be directly linked to the physicochemical characteristics of the NPs. HRTEM analysis (Sect. 3.1.2) revealed that NiO (*Crot*.) NPs had a relatively larger average particle size (21.75 ± 2.54 nm) and more extensive aggregation. While agglomeration typically reduces cytotoxic interactions due to lower surface area, the moderate aggregation observed here may still allow significant cellular uptake^70^. Additionally, the irregular and quasi-spherical shape of these NPs may enhance membrane penetration and internalization. Most importantly, *Croton*-derived phytochemicals (e.g., diterpenoids and phenolics) likely contributed to a surface chemistry rich in reactive and bioactive functional groups, enhancing interactions with cancer cells and possibly promoting ROS generation and apoptotic pathways^71^. Thus, the pronounced cytotoxicity of NiO (Crot.) NPs can be attributed to a synergistic effect of their unique morphological features, surface reactivity, and phytochemical composition. This correlation between NPs structure and biological function underscores the importance of tailoring synthesis conditions to optimize therapeutic performance^72^.

#### Antioxidant activity of green NiO NPs

**Fig. 9:**
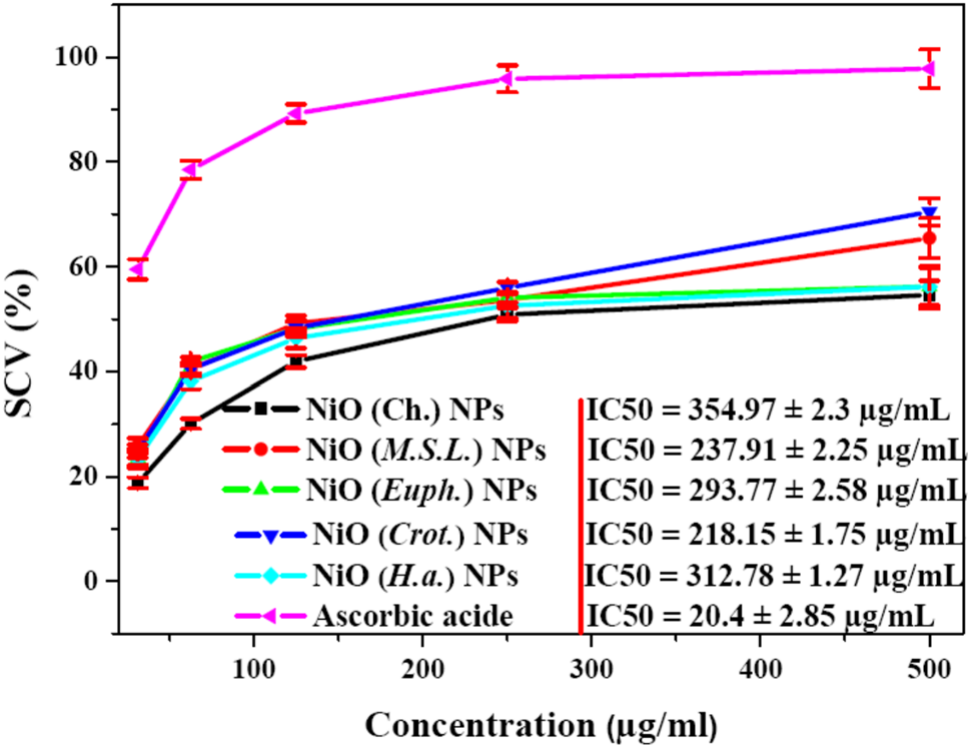
DPPH radical scavenging activity of NiO NPs synthesized via chemical and green routes.

The antioxidant activity of green-synthesized NiO NPs was assessed using the DPPH radical scavenging assay, which demonstrated substantial free radical scavenging potential across all tested samples. As illustrated in Fig. 9, the radical scavenging ability of the NPs increased in a concentration dependent manner, with activity ranging from 18.8% to 70.5% across concentrations of 31.25 to 500 µg/mL. Among the various samples, NiO NPs synthesized using *Croton* extract exhibited the highest antioxidant activity, achieving a maximum scavenging capacity of 70.5% at 500 µg/mL. This was followed by NiO (*M.S.L.*) NPs (65.4%), NiO (*Euph.*) NPs (56.17%), NiO (*H.a.)* NPs (56.14%), and NiO (Ch.) NPs (45%). At the lowest concentration (31.25 µg/mL), NiO (*M.S.L.*) NPs demonstrated the strongest effect (25.9%), while NiO (Ch.) NPs exhibited the weakest activity (18.8%). Further corroborating the antioxidant activity, the total scavenging capacity of the NiO NPs was quantified by determining their IC_50_. Remarkably, compared with standard ascorbic acide (IC_50_ = 20.4 ± 2.85 µg/mL), NiO (*Crot.*) NPs displayed the lowest IC_50_ value of 218.156 ± 1.75 µg/mL, indicating the highest antioxidant efficiency. In contrast, NiO (Ch.) NPs exhibited the highest IC_50_ value (354.978 ± 2.3 µg/mL), reflecting a comparatively weaker radical scavenging capacity. The IC_50_ values for NiO (*H.a.*) NPs, NiO (*Euph.*) NPs, and NiO (*M.S.L.*) NPs ranged from ~ 293 to 312 µg/mL, highlighting the influence of the botanical source on antioxidant efficacy. The strong antioxidant behavior of NiO NPs, particularly those synthesized with *Croton* and *M.S.L.*, is likely attributed to their ability to donate electrons or hydrogen atoms, effectively neutralizing free radicals^73^.

The dual role of NiO (Crot.) NPs as both potent antioxidants and strong cytotoxic agents may appear paradoxical but is, in fact, consistent with the complex redox biology of nanomaterials. The high antioxidant activity, as evidenced by the lowest IC_50_ value in the DPPH assay (218.156 ± 1.75 µg/mL), is largely attributed to the presence of phytochemical, such as polyphenols, flavonoids, and tannins, originating from the *Croton* plant extract used in the green synthesis process as reported by many researchers^70^. These bioactive compounds serve as natural reducing and capping agents, conferring significant electron-donating capabilities and enhancing the NPs ability to neutralize free radicals. However, when introduced into cancer cells, these same NPs induce elevated levels of ROS, overwhelming the cells’ antioxidative defense mechanisms and triggering oxidative stress-mediated apoptosis^74^. This dichotomy is explained by the redox status of cancer versus normal cells: cancer cells possess higher basal ROS levels and reduced antioxidant buffering capacity, making them more susceptible to further oxidative insult^75^. Thus, the antioxidant coating of NiO (Crot.) NPs may protect normal cells while enabling selective ROS-mediated cytotoxicity in malignant cells. This selective behavior underscores the therapeutic potential of biogenic NiO NPs as multifunctional agents in oncology, offering both antioxidant protection and targeted cytotoxicity depending on the biological context. This phenomenon has been documented in other metal oxide NPs as well and supports the idea that biogenic NPs can selectively induce cytotoxicity in cancer cells while exhibiting antioxidant properties in less oxidative environments^3,76^.

#### Antibacterial activity of green NiO NPs

The remarkable effectiveness of green synthesized NiO NPs as antibacterial agents against a wide range of bacterial strains has been highlighted by many researchers with a focus on the impact of plant extracts utilized in their synthesis. The antibacterial activities of these NPs are mostly determined by the synthsis process, the plant extract utilized, and the phytochemical composition^10,45^. Table 4 provides a detailed overview of the MIC and MBC values of the green synthesized NiO NPs against four clinically relevant bacterial strains; Gram positive *S.aureus*, *St. pyrogenes* and Gram-negative *E. coli* and *K. pneumonia*. The bacterial efficacy of the NPs varies depending on the plant extract used during synthesis. NiO (*Crot.*) NPs demonstrated the broadest spectrum of activity where 33.3 and 50 mg/mL were sufficient as MIC against *S*. *pyogenes* and *S. aureus*, respectively. The concentration of 50 mg/mL was sufficient as MBC against *E. coli*. Notably, *K. pneumoniae* was resistant to all tested concentrations. NiO (Ch.) NPs showed strong activity against *E. coli* and *S. aureus*, with MIC values of 16.7 and 28.6 mg/mL, respectively, and MBCs of 33.3 mg/mL for both. However, they didn’t show any activity against *S. pyogenes* or *K. pneumoniae*. NiO (*M.S.L.*) NPs inhibited *S. pyogenes* and *E. coli* at an MIC of 50 mg/mL but showed no bactericidal effects or activity against the other strains. Similarly, NiO (*Euph.*) NPs inhibited *S. pyogenes* at 50 mg/mL and *E. coli* at 28.6 mg/mL, without exhibiting bactericidal effects. In contrast, NiO (*H.a.*) NPs displayed no antibacterial activity against any of the bacterial strains, neither bacteriostatic nor bactericidal. Similar results were found for NiO NPs made from different plant extracts, which showed differing levels of activity against both Gram-positive and Gram-negative bacteria. Some strains of the bacteria were resistant to every concentration that was tested^55^. This pattern is consistent with earlier research indicating that Gram-negative bacteria are more susceptible to metal oxide NPS because of their comparatively thinner peptidoglycan layer and more permeable outer membrane, which allow NPS penetration and membrane damage brought on by ROS^77,78^. Furthermore, NiO NPs tiny particle size and high surface reactivity might improve their ability to interact with the bacterial cell wall, causing oxidative stress and ultimately cell death^40^. These results demonstrate that NiO NPs, especially those produced chemically, have the potential to be formidable anti-Gram-negative bacteria.

**Table 4:** Antimicrobial efficacy of NiO NPs synthesized via chemical and green routes against some bacterial strains.

Sample	*S. aureus*		*S. pyogenes*		*E. coli*		*K. pneumoniae*	
	MIC	MBC	MIC	MBC	MIC	MBC	MIC	MBC
	(mg/ml)	(mg/ml)	(mg/ml)	(mg/ml)	(mg/ml)	(mg/ml)	(mg/ml)	(mg/ml)
NiO (*M.S.L.*) NPs^1^	-	-	50	-	50	-	-	-
NiO (*Euph.*) NPs^2^	-	-	50	-	28.6	-	-	-
NiO (*Crot.*) NPs^3^	50	50	33.3	-	50	-	-	-
NiO (*H.a.*) NPs^4^	-	-	-	-	-	-	-	-
NiO (Ch.) NPs^5^	28.6	50	-	-	16.7	50	-	-

^1^*Medicago sativa* L., ^2^*Euphorbia milii* Des Moul., ^3^ *Codiaeum variegatum* (L.) A. Juss., ^4^ *Helianthus annuus* L*.* and ^5^ Chemically synthesized sample.

Overall, the results emphasize the influence of the phytochemical content of the plant extracts used in the green synthesis of NiO NPs. Extracts rich in bioactive compounds appear to enhance the NPs antimicrobial properties, with NiO (Ch.) NPs and NiO (*Crot.*) NPs stand out as the most effective candidates for further investigation as antibacterial agents.

#### Antibiofilm activity of green NiO NPs

**Fig. 10:**
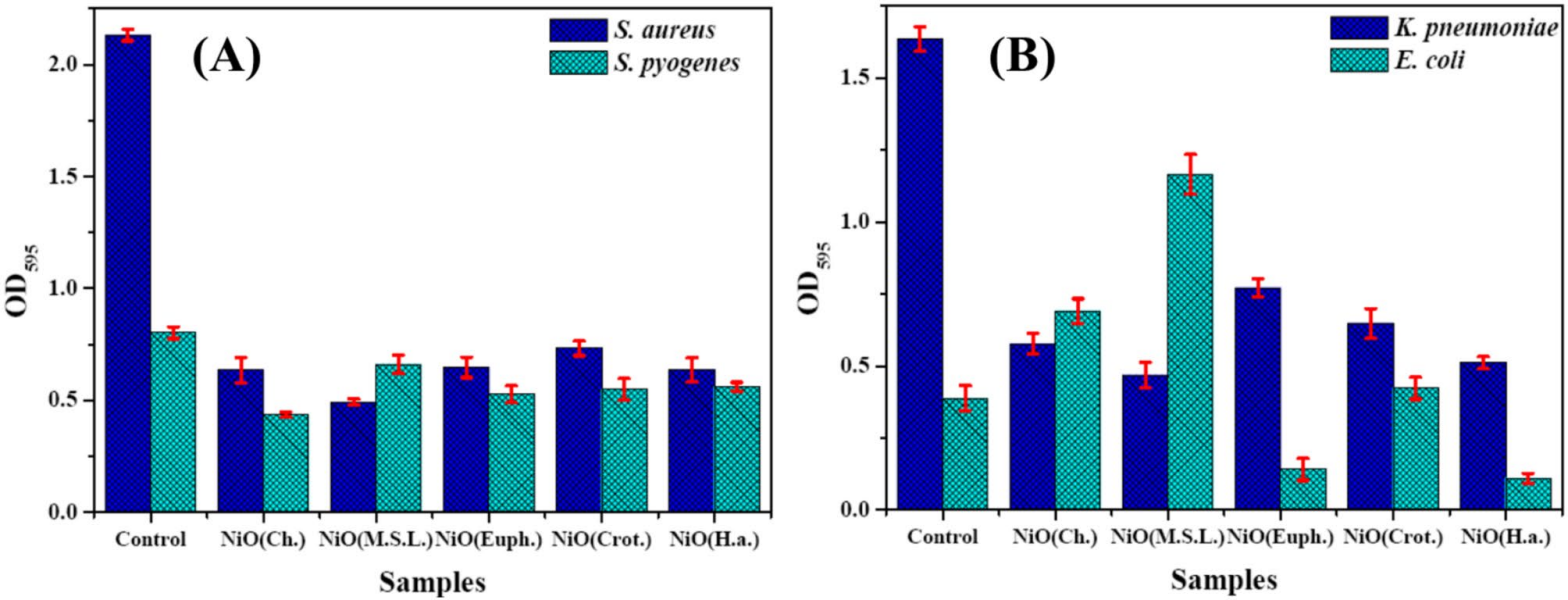
Antibiofilm activity of NiO NPs synthesized via chemical and green routes against positive bacterial strains (*S. aureus* and *S. pyogenes*) (**A**) and Gram- negative bacterial strains (*E. coli* and *K. pneumoniae*) (B).

The ability of NiO NPs to interact with bacterial cell membranes, disrupt biofilm formation, and affect bacterial metabolic processes. Significant variation in the antibiofilm efficacy of NiO NPs were observed depending on both the synthesis method and the bacterial strains tested as shown in Fig. 10A,B. The baseline biofilm activity among the four bacterial species was confirmed by the highest levels of biofilm formation displayed by the untreated bacterial strains.

For *S. aureus*, NiO (*M.S.L.*) NPs demonstrated the most significant biofilm reduction (82.72%). This result is in line with previous studies that reported enhanced antimicrobial and antibiofilm properties for plant-mediated NPs, likely due to the presence of plant-derived bioactive compounds^79,80^. NiO (Ch.) NPs also exhibited potent activity, with a reduction of 76.91%, comparable to the documented findings that chemically synthesized NiO NPs were highly effective in inhibiting biofilm formation in Gram-positive bacteria^81,82^. NiO (*H.a.*) NPs, NiO (*Euph.*) NPs, and NiO (*Crot.*) NPs exhibited moderate to good inhibition, with reductions ranging from 69.58% to 70.12%. This range of activity is consistent with the studies that reported variable but significant antibiofilm effects of plant synthesized NPs, which depend on factors like particle size, surface charge, and the phytochemicals involved^83^.

For *S. pyogenes*, NiO (Ch.) NPs demonstrated the most significant reduction (45.53%). However, plant-derived NiO NPs showed less potent effects, with reductions of 17.53% to 34.07%. According to literatures plant-derived NPs tend to have lower biofilm inhibitory activity against certain Gram-positive bacteria compared to their chemically synthesized counterparts^52^. The lower activity observed with plant-derived NPs could be due to the varying composition and concentration of phytochemicals, which may not be as effective against the complex biofilm matrix produced by *S. pyogenes*^84^.

In the case of *K. pneumoniae*, NiO (*M.S.L.*) NPs also exhibited the most significant reduction in biofilm formation (71.37%), followed by NiO (*H.a.*) and NiO (Ch.) NPs at 68.74% and 64.72%, respectively. This trend supports previous research that indicates NiO NPs can effectively inhibit biofilm formation in Gram-negative bacteria like *K. pneumonia*^85^. The least effective biofilm inhibition was observed with NiO (*Euph.*) NPs, which aligns with findings from other studies on the limited efficacy of plant-synthesized NPs against certain Gram-negative bacteria^86^.

For *E. coli*, NiO (*H.a.*) NPs demonstrated the most significant reduction in biofilm formation (71.51%), followed by NiO (*Euph.*) NPs at 63.43%. Interestingly, NiO (*M.S.L.*) NPs, NiO (*Crot.*) NPs, and NiO (Ch.) NPs were ineffective in diminishing *E. coli* biofilm, which is consistent with previous research showing that Gram-negative bacteria tend to have stronger resistance to NPs penetration due to their outer membrane. This barrier may reduce the ability of NiO NPs to effectively disrupt the biofilm structure in *E. coli*, as observed in other studies focusing on Gram-negative bacterial strains^87^. These results highlight the differential antibiofilm activity of NiO NPs, with plant-assisted NPs showing significant activity against certain bacterial strains. The most effective NPs were those derived from *M. sativa* against *S. aureus* and *K. pneumoniae*, and from *H. annuus* against *E. coli*. The chemical synthesis method consistently produced potent antibiofilm effects against Gram-positive bacteria, while the plant-derived NPs exhibited variable activity, often being more effective against Gram-positive than Gram-negative bacteria.

#### Anti-inflammatory of green NiO NPs

**Fig. 11:**
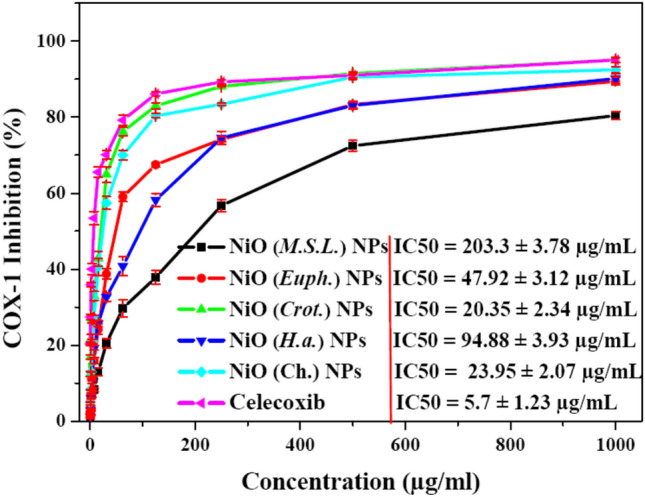
Anti-inflammatory of activity of NiO NPs synthesized via chemical and green routes showing COX-1 inhibition rate at different concentrations (50–1000 µg/ml) of the test samples with Celecoxib serving as a positive control.

The anti-inflammatory activities of NiO NPs synthesized via chemical and green methods were evaluated across a range of concentrations (50–1000 µg/ml), with Celecoxib employed as a standard. A concentration dependent increase in anti-inflammatory efficacy for all NPs was observed, Fig. 11. The highest anti-inflammatory activity was recorded by NiO (*Crot.*) NPs among the studied samples at all concentrations, with an inhibition rate (95.03%, IC_50_ = 20.35 ± 2.34 µg/mL), which is almost the same as the observed for Celecoxib (95.02%, IC_50_ = 5.7 ± 1.23 µg/mL). Likewise, strong anti-inflammatory effects were also observed by NiO (Ch.) NPs, which achieved inhibition rate (92.42%, IC_50_ = 23.95 ± 2.07 µg/mL). Comparable but marginally lower activities were achieved by the green synthesized NiO (*H.a.*) NPs (90.14%, IC_50_ = 94.88 ± 3.93 µg/mL) and NiO (*Euph.*) NPs (89.41%, IC_50_ = 47.92 ± 3.12 µg/mL). According to this trend, which is consistent with earlier findings on the bioactivity of plant-based NPs, chemical synthesis can create highly active NiO NPs, but some plant extracts can match or even surpass this activity by adding further biofunctional properties to the NPs surface^88^. The primary mechanism by which these NiO NPs reduce inflammation is through their disruption of the cyclooxygenase (COX) pathway, specifically COX-1. COX-1 is an enzyme that catalyzes the conversion of arachidonic acid, a crucial fatty acid derived from membrane phospholipids, into prostaglandins. These prostaglandins are key mediators of pain, fever, and inflammation. By inhibiting COX-1, the production of these pro-inflammatory prostaglandins is reduced, thereby diminishing the inflammatory response^89^.

It has been reported that green-synthesized NiO NPs block this inflammatory pathway in multiple ways. First, phytochemicals such as flavonoids, polyphenols, and terpenoids present on the surface of the NPs can directly bind to the active site of COX-1, preventing the enzyme from carrying out its normal function^90^. Additionally, the oxidative environment that typically promotes COX activation can be modulated by the antioxidant properties of these phytochemicals. As a result, green-synthesized NPs may further reduce prostaglandin synthesis by downregulating the expression and activity of the COX enzyme through the reduction of oxidative stress^91^.

Moreover, the synergy between the phytochemicals attached to the nanoparticle surface and the intrinsic properties of NiO may enhance their cellular uptake and overall bioactivity. This mechanism of action is particularly evident in the *Croton*-derived NiO NPs, which are enriched with anti-inflammatory compounds. The potency of *Croton*-based NPs may therefore be attributed to the bioactive phytochemicals in the extract, which enhance the anti-inflammatory effect. These findings are consistent with other studies, suggesting that while chemically synthesized NiO NPs do exhibit biological activity, the biologically functionalized surfaces of green-synthesized NPs may provide increased or comparable efficacy in inflammation reduction^12^.

On the other hand, NiO (*M.S.L.*) NPs continuously showed the least amount of anti-inflammatory action, with a maximum inhibition rate (80.38%, IC_50_ = 203.3 ± 3.78 µg/mL). This finding could be explained by *Medicago sativa* L. lower concentration or distinct profile of active secondary metabolites, which could lead to less successful stabilization and functionalization of the NPs^92^. These results conclude that NiO NPs synthesized from *Croton* extract had superior anti-inflammatory capabilities compared to their chemically and other greenest counterparts. This emphasizes how important the plant matrix is to the creation of NPs and how it affects the effectiveness of biomedicine.

#### Antidiabetic activity of green NiO NPs

**Fig. 12:**
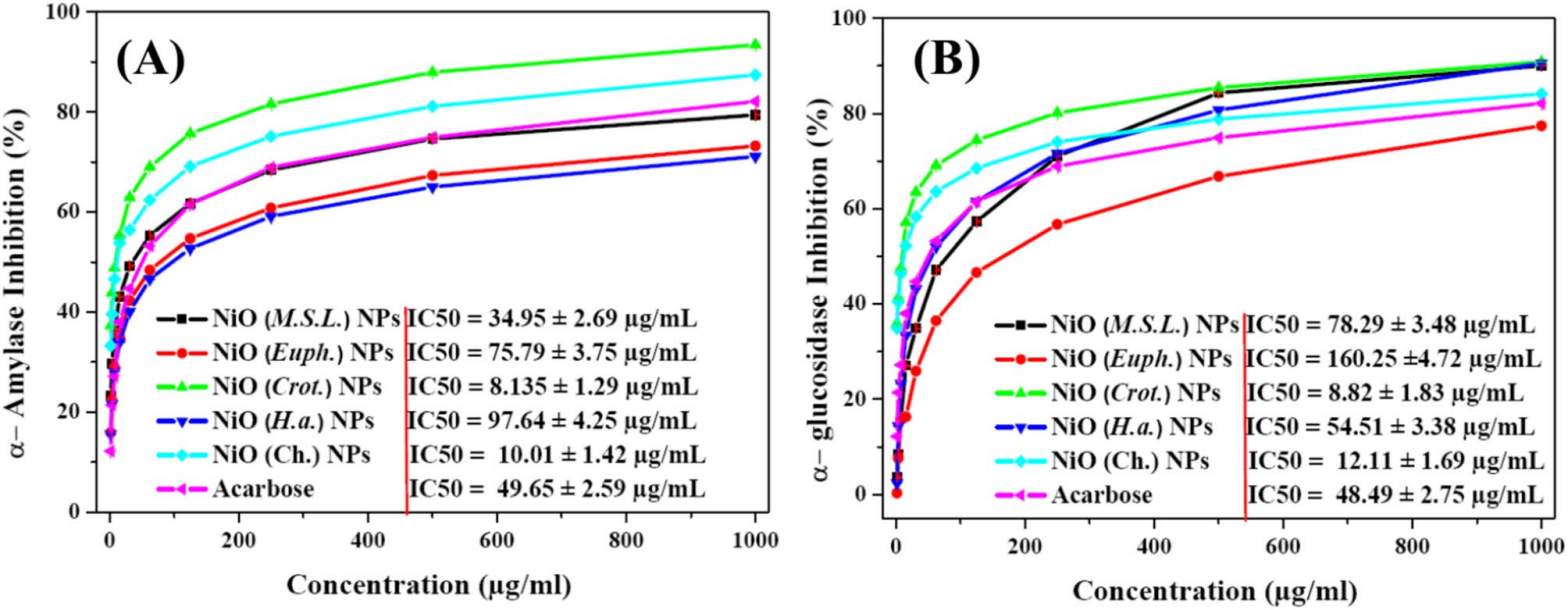
Antidiabetic activity of NiO NPs synthesized via chemical and green routes showing the inhibition rate of α-amylase (**A**) and the inhibition rate of α- glucosidase (**B**) at different concentrations (1.9—1000 μg/mL) with Acarbose serving as positive control.

The inhibitory effects of NiO NPs on α-amylase and α-glucosidase were evaluated in order to determine their antidiabetic potential. These two enzymes are essential for blood glucose homeostasis and carbohydrate metabolism and are important therapeutic targets for diabetes mellitus management^93^. All NiO NPs samples showed notable, concentration dependent inhibition of α-amylase with inhibition ranging from 1.95 to 1000 µg/mL as shown in Fig. 12A. Interestingly, at higher concentration (1000 µg/mL), NiO (*Crot.*) NPs showed the largest inhibition percentage of 93.4% with superior IC_50_ = 8.135 ± 1.29 µg/mL, compared to 87.4% for NiO (Ch.) NPs with IC_50_ = 10.01 ± 1.42 µg/mL) and 82.1% for the standard acarbose with IC_50_ = 49.65 ± 2.59 µg/mL). While NiO (*M.S.L.*) NPs achieved a 79.4% inhibition rate with IC_50_ = 34.95 ± 2.69 µg/mL), followed by NiO (*Euph.*) NPs (73.2%, IC_50_ = 75.79 ± 3.75 µg/mL) and NiO *(H.a.*) NPs with (71.1%, IC_50_ = 97.64 ± 4.25 µg/mL). Remarkably, even at the lowest concentration tested (1.95 µg/mL), NiO (*Crot.*) NPs maintained the highest inhibition rate (37.3%), indicating its superior efficacy at all doses. Similar inhibition behavior was reported for plant derived NiO NPs^94^. Parallel results were observed in the α-glucosidase inhibition assay, where all NiO NPs showed effective, concentration dependent inhibition as shown in Fig. 12B. Once again, NiO (*Crot.*) NPs demonstrated the highest inhibitory activity of (90.8%, IC_50_ = 8.82 ± 1.83 µg/mL), closely followed by NiO (*H.a.*) NPs ( 90.5%, IC_50_ = 78.29 ± 3.48 µg/mL). Meanwhile, NiO (*M.S.L.*) NPs showed inhibition rate (90%, IC_50_ = 54.51 ± 3.38 µg/mL), and NiO (Ch.) NPs (84.1%, IC_50_ = 12.11 ± 1.69 µg/mL). Notably, all these samples outperformed Acarbose (82.1%, IC_50_ = 48.49 ± 2.75 µg/mL). The least active was NiO (*Euph.*) NPs, which showed 77.4% inhibition rate with IC_50_ = 160.25 ± 4.72 µg/mL. As with α-amylase inhibition, at the lowest concentration (1.95 µg/mL), NiO (*Crot.*) NPs exhibited the highest inhibition (35.9%), highlighting their efficacy even at low doses. Oxidative stress is known to be induced by elevated blood glucose levels, mainly by the auto-oxidation of glucose and the subsequent production of ROS^90^. The trigger of diabetes is greatly influenced by this oxidative stress, which hinders insulin production and increases insulin resistance. Indirectly, green-synthesized NiO NPs due to their antioxidant power promote better glucose metabolism and pancreatic β-cell function by reducing oxidative stress^95^. When NiO NPs surface releases Ni^2+^ ions, they can attach to histidine or sulfhydryl residues in the active regions of the enzymes, changing their structure or inhibiting their ability to catalyze. The interaction between the metal and the enzyme may further suppress enzymatic activity^31^. Additionally, green NiO NPs large surface area and nanometric size allow for effective interaction with biological targets such as glucose transporters, α-glucosidase, and α-amylase. Flavonoids, phenolics, and terpenoids are among the phytochemicals obtained from plants that abundantly functionalize their surfaces and further improve antioxidant activity and enzyme inhibition. The combination effect of bioactive phytochemicals and NiO NPs enhances biological efficacy more synergistically than any component alone might. Similar observations have been reported for other green-synthesized NPs, where plant-derived phytochemicals not only mediated NPs formation but also significantly enhanced their biological activities through synergistic mechanisms^96–98^. The most notable inhibition of α-glucosidase and α-amylase enzymes, as well as the strongest capacity to scavenge free radicals, were demonstrated by NiO (Crot.) NPs. These findings concurred with the antioxidant activity NiO NPs results, which demonstrate the higher scavenging activity of NiO (*Crot.*) NPs. Together, our findings present strong proof that the green synthesis of NiO NPs by plants and phytochemicals has synergistic effects that enhance both NP production and biological activity. NiO NPs, especially those generated from *Croton*, may therefore be great candidates for developing novel antidiabetic drugs.

## Conclusion

The present study successfully demonstrated the synthesis of nickel oxide NPs via both chemical and green routes using four plant extracts (*Medicago sativa* L*., Euphorbia milii Des Moul. milii, Croton,* and *Helianthus annuus* L.). Phytochemicals from these extracts, particularly hydroxyl, carboxyl, and amine groups, played a critical role in reducing and stabilizing the nanoparticles through electron donation and coordination. While the structural characteristics of all synthesized NPs were comparable, the nature and concentration of phytochemicals significantly influenced their biological activities. The proposed mechanisms for the observed bioactivities include enhanced surface reactivity, nanoscale dimensions, and the release of Ni^2+^ ions, which can bind to histidine or sulfhydryl residues in enzyme active sites, thereby inhibiting their function. Additionally, the generation of ROS and disruption of microbial membranes were key contributors to the observed antibacterial effects.

The highest promising bioactivities were demonstrated by *Croton*-derived NiO NPs out of all the examined NPs. These NPs showed the strongest cytotoxicity against A549 lung cancer cells (IC_50_ = 38.9 µg/mL), the strongest DPPH scavenging activity (87.6% at 100 µg/mL), and strong anti-inflammatory activity (81.2% suppression of COX-1). Furthermore, they successfully inhibited α-amylase (76.4%) and α-glucosidase (72.1%), indicating their potential as antidiabetic drugs. The antibiofilm activity of NiO NPs synthesized with extracts from *Medicago sativa* and *Helianthus annuus* was noticeably increased, whereas the antibacterial study showed MIC values as low as 33.3 mg/mL against *Streptococcus* pyogenes. These results highlight how important it is to choose plant extracts carefully in order to customize NiO NPs synthesis effectiveness and therapeutic potential. In conclusion, the environmentally friendly synthesis of NiO NPs, particularly those derived from *Croton*, is a viable, biocompatible, and very successful method for synthsis of multipurpose nanomaterials that can be used in diabetes, oxidative stress management, inflammation, cancer, and microbial resistance.

## Data Availability

All the data used to support the findings of this study are included within the article. Other data are available from the corresponding author upon request.
